# A comparison of nonlinear mixed models and response to selection of tick-infestation on lambs

**DOI:** 10.1371/journal.pone.0172711

**Published:** 2017-03-03

**Authors:** Panya Sae-Lim, Lise Grøva, Ingrid Olesen, Luis Varona

**Affiliations:** 1 Nofima AS, Osloveien 1, Ås, Norway; 2 Norwegian Institute of Bioeconomy Research (NIBIO), Gunnars veg 6, Tingvoll, Norway; 3 Faculty of Veterinary, University of Zaragoza, Zaragoza, Spain; University of Minnesota, UNITED STATES

## Abstract

Tick-borne fever (TBF) is stated as one of the main disease challenges in Norwegian sheep farming during the grazing season. TBF is caused by the bacterium *Anaplasma phagocytophilum* that is transmitted by the tick *Ixodes ricinus*. A sustainable strategy to control tick-infestation is to breed for genetically robust animals. In order to use selection to genetically improve traits we need reliable estimates of genetic parameters. The standard procedures for estimating variance components assume a Gaussian distribution of the data. However, tick-count data is a discrete variable and, thus, standard procedures using linear models may not be appropriate. Thus, the objectives of this study were twofold: 1) to compare four alternative non-linear models: Poisson, negative binomial, zero-inflated Poisson and zero-inflated negative binomial based on their goodness of fit for quantifying genetic variation, as well as heritability for tick-count and 2) to investigate potential response to selection against tick-count based on truncation selection given the estimated genetic parameters from the best fit model. Our results showed that zero-inflated Poisson was the most parsimonious model for the analysis of tick count data. The resulting estimates of variance components and high heritability (0.32) led us to conclude that genetic determinism is relevant on tick count. A reduction of the breeding values for tick-count by one sire-dam genetic standard deviation on the liability scale will reduce the number of tick counts below an average of 1. An appropriate breeding scheme could control tick-count and, as a consequence, probably reduce TBF in sheep.

## Introduction

Sheep farming in Norway is based on grazing unfenced rangeland and mountain pastures in summer. Grazing such pastures do however provide welfare and production challenges with losses to predators, diseases and accidents. There is a yearly national economic loss of approximately 100 million NOK (125,000 sheep and lambs lost every year) while it is also a severe animal welfare issue [1,2].

Tick-borne fever (TBF) is stated as one of the main disease challenges in Norwegian sheep farming during the grazing season, particularly along the coast of south-western Norway [3]. It is proposed to be an explanatory factor of the observed increase in lamb loss during the last decades [4]. TBF is caused by the bacterium *Anaplasma phagocytophilum*, an intragranulocytic alpha-proteobacterium belonging to the family *Rickettsiaceae*, that is transmitted by *Ixodes* ticks [5, 6, 7]. *A*.*phagocytophilum* infects neutrophils and survives for several months by avoiding bacteriacidal defence mechanisms in immune-competent sheep [8, 9]. Lamb losses as high as 30% in a flock due to *A*. *phagocytophilum* infection are reported [10] and the Norwegian Food Safety Authority considers restrictions of grazing on pastures with high losses due to the severe welfare problems. Ticks and tick-borne diseases have, for a long time, also been a major concern to livestock producers in tropical and sub-tropical regions of the world [11, 12, 13].

Tick-infestation in sheep is commonly controlled by chemical treatment or insecticide, known as acaricides. The frequent use of such treatment is however costly and associated with development of resistance against such treatment, particularly in one-host ticks [14, 15]. An alternative strategy to control tick-infestation is to breed for genetically resistant or more robust animals. Theoretical arguments are raised as potential problems in selection for resistance, but a number of studies support that it can be sustainable, feasible and desirable [16]. Genetic variation in resistance is shown in many farmed species, where numerous diseases are involved [17] i.e., gastrointestinal nematode infections [18], mastitis [19], foot rot [20], ectoparasites, i.e., flies and lice [21, 22] and scrapie [23] in sheep. Various levels of host resistance to tick-infestation are found to occur in different breeds of cattle and have been implemented in breeding schemes [24–26]. Individual variation in response to *A*. *phagocytophilum* infection in sheep is evident and shown by Granquist et al. [27] and Stuen et al. [28]. Furthermore, genetic variation in lamb survival on tick exposed pastures has also been reported [29]. This variation in response to infection might include genetic variation in resistance or susceptibility to tick-borne infections. The risk of being infected is likely to increase with number of ticks infested as approximately 8.8% of the ticks are infected with the bacterium [30]. Hence, tick-count on lambs may, to some degree, reflect the susceptibility of *A*. *phagocytophilum* infection in lambs. However, to our knowledge, genetic variation of tick-count of the *Ixodes ricinus* on sheep has not been studied. For cattle, host resistance to ticks has been shown to be under genetic control [31] and tick count is suggested to be an appropriate method to measure tick resistance or susceptibility [32, 33]. Resistance of cattle to ticks is heritable and responsive to selection [34] and heritability estimates for resistance to ticks on cattle range from 0.05 to 0.42 [35–37].

In order to use selection to genetically improve traits we need reliable estimates of genetic parameter. The standard procedures using linear models for estimating variance component assume a Gaussian distribution of the data [38]. However, tick-count data is a discrete variable and, thus, such standard procedures may not be appropriate. One potential alternative could be to use nonlinear or generalized linear models with discrete link functions, such as the Poisson or negative binomial distributions. Data with a Poisson distribution have equal mean and variance whereas negative binomial allows the data to be overdispersed. Moreover, the nature of tick-count data expresses a higher-than-expected incidence of zero scores, but this can be accommodated with zero-inflated versions [39–41] of the above described distributions.

In addition, response to selection for tick-count in sheep has not been studied, and our effort is to study the potential of selection for tick-count to consider selecting for it in a breeding program. So far, only one theoretical study on prediction of response to selection for Poisson distributed traits is published [42]. Thus, such prediction may deviate from the response to the truncation selection assuming the estimated genetic effects here or when the distribution of tick-count data deviates from the conventional Poisson distribution.

Therefore, the objectives of this study were two folds: 1) to compare four different statistical models, i.e., Poisson, negative binomial, zero-inflated Poisson, and zero-inflated negative binomial based on the their goodness of fit for quantifying within-breed genetic variation, as well as, heritability for tick-count in lambs on tick-infested pastures, and 2) to investigate potential response to selection against tick-count based on truncation selection given the estimated genetic parameters from the best fit model.

Furthermore, following Foulley and Im [43]’s methodology, we derived a heritability (observed scale) while accounting for additional random effects in the non-linear model which it has not been accounted for when deriving the heritability in the previous study [43].

## Materials and methods

### Ethics statement

All procedures were in accordance with ethical standards as regulated by Regulation for use of animals in trials (FOR-2015-06-18-761) which is in accordance with EU directive 2010/63/EU on protection of animals that are used for scientific purposes. The regulation is administrated by the Norwegian Food Safety Authorities (www.mattilsynet.no). The procedure of counting ticks on lambs did not need official approval as of current regulations at start of study. All sheep included in the study were managed, handled and cared for in the same manner as in regular sheep production systems. Catching and holding sheep for examination is a common and normal procedure in sheep production, e.g. for shearing, treatment, health check. Counting ticks on sheep did not cause any additional pain or discomfort apart from the routine care of being caught and held for visual examination. Handling sheep for tick count was conducted by experienced sheep handlers to keep handling stress at a minimum. The sheep handlers were not certified veterinarians, but experienced sheep handlers.

### Data source

**Fig 1:**
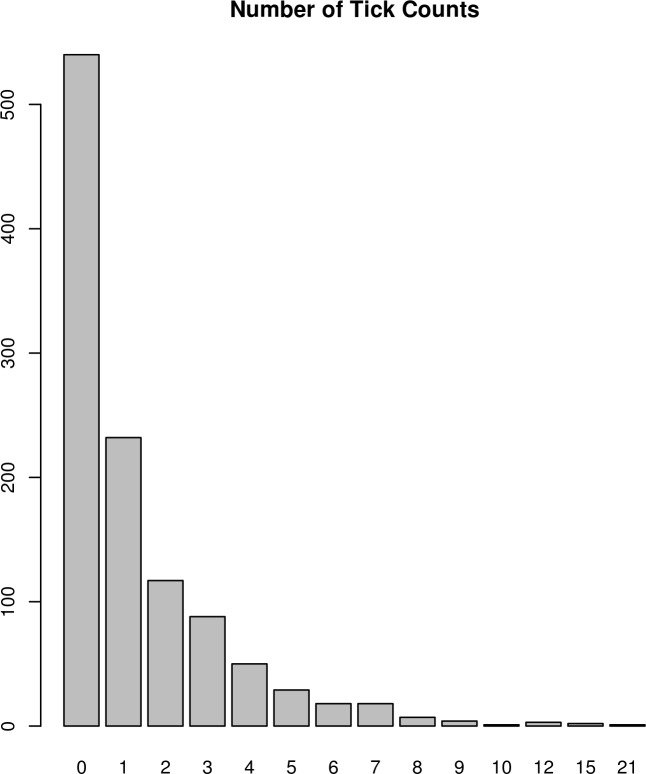
Histogram of number of tick counts.

The study was conducted in 2011, 2012 and 2013 on 6 sheep farms in tick endemic areas on the west coast of Norway in Vestnes (62°37'16.5"N 7°05'23.0"E), Rauma (62°35'15.2"N 7°41'10.0"E) and Fræna (62°51'13.3"N 7°09'14.5"E) municipalities where ticks and losses to TBF had been observed earlier. Presence of ticks was confirmed by examining the pastures for questing ticks with the cloth lure method [44] at the same day as counting ticks on the lambs. The lambs and their mothers were not treated with acaricides until after the last tick count was conducted. Tick counts were recorded on 555 lambs (Norwegian White Sheep breed) grazing on 12 different fenced pastures during spring. Lambs were sired by 87 rams that were mated with 283 dams. Tick-counts were repeatedly recorded once on the same lambs on approximately 1 and 2 weeks after turn out to pasture from the winter indoor feeding period. Lambs were turned out on spring pasture between 2–18 May on the various farms and pastures in the study. First tick count was conducted 7 days after turn out on spring pasture; i.e. between 9–25 May. Second tick count was conducted 6–7 days after first tick count; i.e. 16 May–1 June. Ewes and lambs were turned out on summer range pasture in early June for approximately 12–15 weeks. Tick counts were observed on the head, armpit, and groin of the lamb. The average number (and standard deviation) of tick counts were 1.35 (2.06). The distribution of ticks on the different sites and body locations is presented in Table 1. The raw distribution of tick counts is presented in Fig 1, ranging between 0 and 21, with 49% of 0 tick, 46% of 1–5 ticks, and 5% of observations with greater than 5 ticks.

**Table 1:** Average and its standard deviation (SD) tick count per lamb per farm and pasture.

Farm	Pasture	n	Tick count	
			First	Second
A	1	37	0.81 ^1.02^	0.32 ^0.47^
A	2	22	0.41 ^0.66^	0.41 ^0.59^
B	3	40	0.58 ^0.84^	0.58 ^0.78^
C	4	60	1.65 ^1.27^	2.20 ^1.89^
D	5	39	0.13 ^0.41^	0.02 ^0.16^
D	6	42	0.21 ^0.42^	0.19 ^0.51^
D	7	34	0.08 ^0.51^	0.03 ^0.17^
E	8	129	1.45 ^2.29^	0.88 ^1.77^
F	9	37	0.76 ^0.72^	0.51 ^0.73^
F	10	34	3.44 ^2.19^	2.44 ^1.42^
F	11	37	4.11 ^3.18^	3.00 ^1.72^
F	12	44	4.77 ^3.67^	2.57 ^1.65^

n = number of observations, two repeated tick counts were registered (the first and the second), the superscripts = SD.

### Genetic analysis

#### Models and posterior distributions

Data were analysed using Poisson and negative binomial sire-dam models and their zero-inflated versions. Zero-inflated models assumed the data were generated from a mixture of two distributions although the population membership was not observed. The first level of the hierarchy, the probability mass function of the response *Y*_i_ (tick-count) was:

$$\mathrm{Pr}\left(Y_{i}=y_{i}|\eta ,\theta_{i}\right)=\{\begin{array}{c} \eta +f\left(Y_{i}=0|\theta_{i}\right)\left(1-\eta \right),\;y_{i}=0,\\ f\left(Y_{i}=y_{i}|\theta_{i}\right)\left(1-\eta \right),\;y_{i}=1,2,\ldots ..\;0\leq \eta \leq 1 \end{array}$$ (1)

where *Y*_i_ has a probability mass function corresponding to a Poisson or negative binomial distribution defined by a set of parameters *θ*_*i*_, which is assigned with a probability (1 − *η*) and a degenerate distribution supported at zero with probability *η*. Further, the standard or non zero-inflated version of the models is generated by setting *η* = 0.

The mean and the variance of the zero-inflated are:

$$E\left(Y_{i}|\eta ,\theta_{i}\right)=\left(1-\eta \right)E\left(Y_{i}|\theta_{i}\right),$$ (2)

and

$$Var\left(Y_{i}|\eta ,\theta_{i}\right)=\left(1-\eta \right)Var\left(Y_{i}|\theta_{i}\right)+\eta \left(1-\eta \right){\left[E\left(Y_{i}|\eta ,\theta_{i}\right)\right]}^{2}$$ (3)

For the Poisson model *θ*_*i*_ = (*λ*_*i*_), the probability mass function is:

$$f\left(Y_{i}=y_{i}|\lambda_{i}\right)=\frac{\lambda_{i}^{y_{i}}\exp\left(-\lambda_{i}\right)}{y_{i}!},\;\lambda >0,\;Y_{i}=0,1,\ldots .,$$ (4)

with mean and variance *E*(*Y*_*i*_|*λ*_*i*_) = *Var*(*Y*_*i*_|*λ*_*i*_) = *λ*_*i*_.

For the negative binomial model *θ* = (*ω*, *κ*) and the probability mass function is:

$$f\left(Y_{i}=y_{i}|\omega ,\kappa \right)=\frac{\Gamma \left(\kappa +y_{i}\right)}{\Gamma \left(\kappa \right)y_{i}!}{\left(\frac{\omega }{\kappa +\omega }\right)}^{y_{i}}{\left(\frac{\kappa }{\kappa +\omega }\right)}^{\kappa },$$ (5)

where *ω* is the mean and *κ* is the shape parameter which approaches to the Poisson distribution as *κ* → ∞, and with *E*(*Y*|*ω*_*i*_, *κ*) = *ω*_*i*_ and $var(Y|\omega_{i},\kappa )=\omega_{i}+\frac{\omega_{i}^{2}}{\kappa }$.

At the second level of hierarchy, we define $\ln\;\lambda ={\left\{ln\;\lambda_{i}\right\}}_{i=1}^{n}$ and $\ln\;\omega ={\left\{\ln\;\omega_{i}\right\}}_{i=1}^{n}$ for the Poisson and negative binomial model respectively. Further *n* is the number of tick count observations. The underlying linear model for ln *λ* and ln *ω* are:

$$\ln\lambda =Xb_{\lambda }+\left(Z_{\text{s}}+Z_{\text{d}}\right)u_{\lambda }+Wp_{\lambda }+Tm_{\lambda },$$ (6)

and

$$\ln\omega =Xb_{\omega }+\left(Z_{\text{s}}+Z_{\text{d}}\right)u_{\omega }+Wp_{\omega }+Tm_{\omega },$$ (7)

where **b**_*λ*_(**b**_*ω*_) is the vectors of fixed systematic effects (record-farm and pasture-farm with 12 levels each). The **u**_*λ*_(**u**_*ω*_), **p**_*λ*_(**p**_*ω*_), and **m**_λ_(**m**_ω_) are the vectors of sire-dam genetic random effects, permanent environmental random effects and maternal environmental random effects, respectively. The **X**, **Z**_s_, **Z**_d_, **W** and **T** are the corresponding incidence matrices. The link function depends on the distribution of the parameters: Poisson, negative binomial, or the zero-inflated version of those. Prior distributions for **u**, **p** and **m** were the following multivariate Gaussian (MVN) distributions:

$$u∼MVN\left(0,A\sigma_{u}^{2}\right),\;p∼MVN\left(0,I\sigma_{p}^{2}\right)\;\mathrm{and}\;m∼MVN\left(0,I\sigma_{m}^{2}\right).$$

In addition prior distributions of the variance components and systematic effects was bounded uniform. Moreover, prior distributions of *κ* in the negative binomial model was also assumed bounded uniform. Finally, the prior distribution for *η* in the zero-inflated models was uniform between 0 and 1.

#### Markov chain Monte Carlo (McMC)

Models were implemented using a Gibbs Sampler **McMC** algorithm [45] that involved an updating sampling scheme from the conditional posterior distributions of all the unknowns in the model. Here, conditional posterior distributions of location parameters (**b**, **u**, **p** and **m**) where unknown and single-site Metropolis-Hasting updates with random walk proposal were implemented [46]. Further, conditional posterior distributions for variance components were scaled inverted chi-square distributions. The Gibbs sampler was implemented with a single long chain of 1,250,000 after discarding the first 250,000. Convergence of the **McMC** chains was explored by visual inspection of the chain.

#### Goodness of fit statistics

Models were compared by means of the pseudo log-marginal probability of data (logCPO) and the Deviance Information Criterion (DIC).

*Log-marginal probability (logCPO)*: If we consider the data vector **y** = (*y*_*i*_,**y**_−*i*_), where *y*_i_ is the *i*^th^ datum and **y**_-i_ is the vector of data with *i*^th^ datum deleted, the conditional predictive distribution has a probability density equal to:

$$p\left(y_{i}|y_{-i}\right)=\int p\left(y_{i}|y_{-i}\right)f\left(\theta |y_{-i}\right)dy,$$ (8)

where *θ* is the vector of parameters. Therefore, *p*(*y*_*i*_|**y**_−*i*_) can be interpreted as the probability of each datum given the rest of the data, and it is known as the conditional predictive ordinate (CPO) for the *i*^th^ datum. The pseudo log-marginal probability of the data is then:

$$\sum_{i}\ln\;p\left(y_{i}|y_{-i}\right).$$ (9)

A Monte Carlo approximation of the logCPO suggested by Gelfand [47] is:

$$\sum_{i}\ln\;\hat{p}\left(y_{i}|y_{-i}\right),$$ (10)

where

$$\hat{p}\left(y_{i}|y_{-i}\right)=N{\left[\sum_{j=1}^{N}\frac{1}{p\left(y_{i}|\theta^{j}\right)}\right]}^{-1},$$ (11)

and *N* is the number of the **McMC** draws, and *θ*^*j*^ is the *j*^th^ draw from the posterior distribution of the corresponding parameter. The higher the value of the LogCPO, the better the model fit to the data.

*Deviance Information Criterion*: The Deviance Information Criterion (DIC) was defined by Spiegelhalter et al. [48]. It compares the global quality of 2 or more models accounting for model complexity. For a particular model *M*, the DIC is defined as:

$$DIC_{M}=2{\bar{D}}_{M}-D\left({\bar{\theta }}_{M}\right),$$ (12)

where ${\bar{D}}_{M}$ is the posterior expectation of the deviance *D*(*θ*_*M*_), and $D({\bar{\theta }}_{M})=-2log(p(y|\theta_{M}))$ is the deviance evaluated at the posterior mean estimate of the parameter vector (*θ*_*M*_). The computation of DIC is composed by two terms, i.e., ${\bar{D}}_{M}$ is a measure of model fit and ${\bar{D}}_{M}-D({\bar{\theta }}_{M})$ is related with the effective number of parameters. Models with smaller DIC exhibit a better global fit after accounting for model complexity. Differences in DIC of more than 7 units are considered important by Spiegelhalter et al. [48].

#### Calculation of genetic parameters

For the sire-dam model, the estimated variance component for sires was equal to the variance component for dams and equal to one quarter of the additive genetic variance or $\sigma_{u}^{2}=\sigma_{sd}^{2}=\frac{1}{4}\sigma_{a}^{2}$. Therefore, the additive genetic variance for tick-count $\left(\sigma_{a}^{2}\right)$ on the liability scale was estimated as $4\sigma_{u}^{2}$.

Further, the phenotypic variance ($\sigma_{y}^{2}$) was calculated, following Foulley and Im [39] as:

$$\sigma_{y}^{2}=E_{\theta }[var(Y|\theta )]+{var}_{\theta }[E(Y|\theta )]$$ (13)

and the residual variance ($\sigma_{r}^{2}$) was calculated as:

$$\begin{array}{l} \sigma_{r}^{2}=E_{\theta }\left[var\left(Y|\theta \right)\right]\\ \sigma_{r}^{2}=\left(1-\eta \right)var\left(Y|\theta \right)+\eta \left(1-\eta \right){\left[E\left(Y|\theta \right)\right]}^{2}\\ \sigma_{r}^{2}=\left(1-\eta \right)var\left(Y\right)+\eta \left(1-\eta \right){\left[E\left(Y\right)\right]}^{2}. \end{array}$$ (14)

Under the Poisson distribution:

E(Y)=var(Y)=λ (15)

And under the negative binomial distribution,

$$E(Y)=\omega_{i}\;\mathrm{and}\;var(Y)=\omega_{i}+\frac{\omega_{i}^{2}}{\kappa },$$ (16)

$$\sigma_{nr}^{2}=var_{\theta }\left[E\left(Y|\theta \right)\right]={\left(1-\eta \right)}^{2}E{\left(Y\right)}^{2}\left[\text{exp}\left(\sigma_{t}^{2}\right)-1\right],$$ (17)

where the *nr* is the non-residual components and the $\sigma_{t}^{2}={2\sigma }_{sd}^{2}+\sigma_{p}^{2}+\sigma_{m}^{2}$.

Finally, and following Foulley and Im [39], the additive genetic variance on the observed scale ($\sigma_{g}^{2}$) can be achieved by:

$$\sigma_{\text{g}}^{2}=\frac{{\left[cov\left(\text{g},a\right)\right]}^{2}}{\sigma_{a}^{2}}=\frac{{\left[\left(1-\eta \right)E\left(Y\right)\sigma_{a}^{2}\right]}^{2}}{\sigma_{a}^{2}}$$ (18)

$$\sigma_{\text{g}}^{2}={\left(1-\eta \right)}^{2}E{\left(Y\right)}^{2}\sigma_{a}^{2}$$ (19)

Therefore, the heritability on the observed scale is computed as:

$$h^{2}=\frac{{\left(1-\eta \right)}^{2}{\left[E\left(Y\right)\right]}^{2}\sigma_{a}^{2}}{\left(1-\eta \right)var\left(Y\right)+\eta \left(1-\eta \right){\left[E\left(Y\right)\right]}^{2}+{\left(1-\eta \right)}^{2}{\left[E\left(Y\right)\right]}^{2}\left[exp\left(\sigma_{t}^{2}\right)-1\right]}$$ (20)

### Response to selection

After the model comparison, the response to selection for tick-count was calculated based on the best-fitted model. In order to quantify the potential application of the procedure for breeding for low tick number, we computed the expected number of ticks for the progeny of the individuals with the bottom 5, 10 and 20% breeding values. For comparison and theoretical interest, top 5, 10 and 20% individuals were also selected. Expected response was calculated for the total population. Given the non-linear nature of the model, calculations were performed for several reference points of average tick number (0.5, 1.0, 1.5, 2.0, 2.5 and 3.0). For each group, we calculated the average posterior mean estimate of the breeding value (${\bar{u}}_{SEL}$) and, later on, the expectation of the progeny of the selected group computed as:

$$E\left(Y_{i}|\eta ,\theta_{i}\right)=\left(1-\eta \right)E(Y_{i}|\theta_{i}),$$ (21)

where *η* is the posterior mean estimate and $\theta_{i}=exp(\log\left(r\right)+{\bar{u}}_{SEL})$, where *r* is the reference point (0.5, 1.0, 1.5, 2.0, 2.5 and 3.0).

## Results and discussion

### Model comparison

The most frequent model for analysis of animal breeding data is the standard linear model [49], or the threshold model for categorical data [50]. In this study, we discarded its implementation given the clear discrepancy with the Gaussian distribution and the wide range of categories of data (Fig 1). Thus, we restricted our comparison to Poisson [51] and negative binomial [52] models and its zero-inflated versions [41,46] using a Bayesian approach. The interpretation of zero-inflated models in the tick count data implies that the records are generated from a mixture of two distributions. First, a Bernoulli distribution that determines whether the individual has been in contact with ticks or not and, second, a Poisson (P) or negative binomial (NB) distribution that affects tick count only if the individual has been in contact with ticks.

The results of the variance component estimation and the model comparison are presented in Table 2. Both LogCPO and DIC criteria indicated that zero-inflated versions were better in goodness of fit than nonzero-inflated ones. Moreover, the zero-inflated Poisson (ZIP) model (logCPO = -1435.33: DIC = 2805.54) fitted the data slightly better than zero-inflated negative binomial (ZINB) model (logCPO = -1435.66: DIC = 2807.58), as the model is slightly more parsimonious avoiding the estimation of the parameter *κ*. In fact, considering the DIC, a reduction of more than 7 units usually indicates a significant improvement of goodness of fit [48]. In this study, we found that ZINB model results in considerably lower DIC by 46.36 units than NB model. Similarly, ZIP model also provides 14.82 lower units of DIC than conventional P model. Hence, the most fitted model in this study was ZIP model.

**Table 2:** Posterior mean (SD) of variance components, heritability over Markov chain Monte Carlo replicates and LogCPO and DIC from different models.

Model	LogCPO	DIC	*κ*	*η*	*λ*	*ω*	$\sigma_{sd}^{2}$	$\sigma_{pe}^{2}$	$\sigma_{m}^{2}$	$\sigma_{g}^{2}$	$\sigma_{nr}^{2}$	$\sigma_{r}^{2}$	*h*^2^
ZINB	-1435.7	2807.6	3102.29 (1634.50)	0.042 (0.016)	n.a.	1.421 (0.042)	0.114 (0.057)	0.097 (0.041)	0.184 (0.083)	0.848 (0.429)	1.225 (0.279)	1.447 (0.051)	0.321 (0.167)
ZIP	**-1435.3**	**2805.5**	n.a.	0.042 (0.016)	1.421 (0.041)	n.a.	0.114 (0.055)	0.099 (0.042)	0.183 (0.083)	0.844 (0.414)	1.229 (0.297)	1.442 (0.047)	0.320 (0.162)
NB	-1441.6	2853.9	12.89 (46.51)	n.a.	n.a.	1.359 (0.042)	0.112 (0.049)	0.059 (0.040)	0.138 (0.071)	0.829 (0.371)	0.969 (0.242)	1.594 (0.105)	0.327 (0.151)
P	-1460.2	2820.4	n.a.	n.a.	1.349 (0.034)	n.a.	0.108 (0.050)	0.114 (0.043)	0.144 (0.074)	0.791 (0.371)	1.114 (0.245)	1.349 (0.034)	0.324 (0.156)

ZINP = zero-inflated negative binomial model, ZIP = zero-inflated Poisson model, NB = negative binomial model, P = Poisson model. LogCPO = logarithm of conditional predictive ordinate, DIC = deviance information criterion, *κ* = shape parameter of negative binomial distribution, *η* = probability of zero-inflated model, *λ* = mean value of the Poisson distribution, *ω* = mean value of the negative binomial distribution. On the liability scale: $\sigma_{sd}^{2}$ = estimate of sire-dam variance, $\sigma_{pe}^{2}$ = estimate of permanent environmental variance, $\sigma_{m}^{2}$ = estimate of maternal environmental variance. On the observed scale: $\sigma_{g}^{2}$ = estimate of additive genetic variance, $\sigma_{nr}^{2}$ = estimate of non-residual variance, $\sigma_{r}^{2}$ = estimate of residual variance, *h*^2^ = heritability. n.a. = not applicable. The bold number indicates the highest goodness of fit to the tick count data.

These results can be confirmed based on the average posterior mean estimates of *λ*, *ω* and *κ* under different models. First, we will compare the P and NB models. The average location parameter of negative binomial distribution (*ω* = 1.359) was very similar to the average Poisson parameter (*λ* = 1.349), but the variance of the negative binomial distribution was greater than *λ* in Poisson distribution as the posterior mean estimate of the *κ* parameter (12.89) was far away from infinity. This indicated the presence of over-dispersion in the dataset. Consequently, based on logCPO, NB model (logCPO = -1441.58) fitted the data better than the P model did (logCPO = -1460.21), indicating that accounting for over-dispersion can improve goodness of fit (Table 2). Although we implemented Bayesian process, our results based on logCPO is in line with the simulation study by Tempelman and Gianola [52] who used marginal maximum likelihood methodology to compare P and NB models and found that estimates under NB model were less biased and yielded lower mean square error. However, DIC in our study indicated the opposite because the Poisson model (DIC = 2820.4) fitted the data better than the NB model (DIC = 2853.9). These contradicting results may be due to the effect of excessive presence of zeroes in tick counts, which it could not be appropriately accounted for with conventional Poisson and NB models. Nevertheless, when the link function takes into account the possibility of an excess of zeroes by the zero-inflated version the distributions, the average posterior estimate of *ω* moved towards *λ* (1.421), while the *κ* increased substantially, suggesting that the variance of the zero-inflated negative binomial distribution was moving closer to the *ω*. This yielded similar distribution to ZIP, and the ZIP model was selected as the best when using logCPO and DIC approaches. Both the ZIP and ZINB models yielded a posterior mean estimate of 0.042 for the probability of zero (*η*), proving that even a low probability of zero ticks improves considerably the goodness of fit of models.

The model comparisons for count data has been studied empirically and reported mainly in cattle and sheep [53–55]. The use of Poisson model was often the first choice for fitting the count data in comparison to a linear or threshold model. Nevertheless, most of the analysis was dedicated to litter size data, and they provide a better adjustment for threshold and linear models than for Poisson, such as in the study of Olesen et al. [55]. However, the raw distribution of tick count data (Fig 1) diverges clearly from the distribution expected for litter size. In fact, the only precedent in analysing tick count data was a study of crossbreed Hereford x Nellore cattle [54] suggesting that the Poisson model fits better than linear model. However, there was also a clear excess of zeroes (48.6%) in that data set, indicating that zero inflated distributions could have performed even better as shown by the model comparison analysis described above.

We also performed some additional model comparisons with the same models (P, NB, ZIP and ZINB) while fixing $\sigma_{sd}^{2}=0$ in order to determine the genetic determinism of tick count. The results are presented in S1 Table. We found that DIC decreased by 7.24 units for P model, 12.38 units for NB model, 6.82 units for ZIP model and 7.42 units for ZINB and also the logCPO estimates were worst for the model with null sire-dam genetic variation. These results confirmed that $\sigma_{sd}^{2}$ was relevant and different from zero for tick counts in lambs.

### Genetic parameters

**Fig 2:**
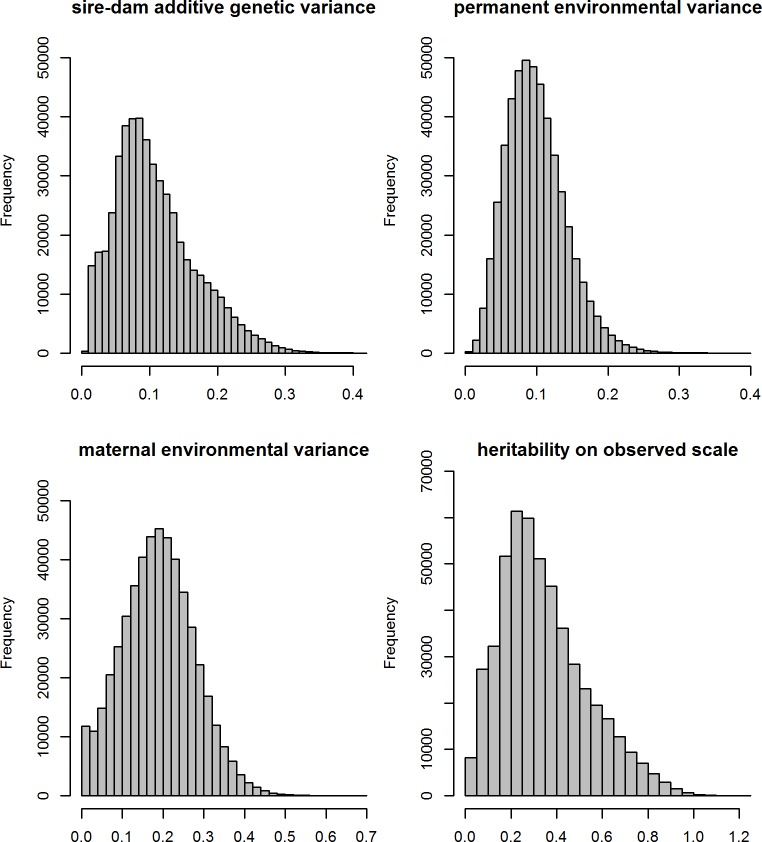
Posterior distribution of the estimates of sire-dam variance, permanent environmental variance, maternal environmental variance, and heritability on the observed scale.

Different models did neither noticeably influence the estimates of variance components on the observed nor the liability scales (Table 2). In Fig 2, the posterior distributions of the variance components ($\sigma_{sd}^{2},\sigma_{m}^{2},\sigma_{pe}^{2}$) and heritability of the observed scale calculated using Eq 20 and for the selected model (ZIP) are presented. The average of $\sigma_{sd}^{2}$ over **McMC** samples was 0.114 with a standard deviation of 0.055, confirming the results of the model comparison with the reduced models ($\sigma_{sd}^{2}=0$) presented above. At the liability scale, the estimate of additive genetic variance ($\sigma_{a}^{2}=4\sigma_{sd}^{2}$) under model ZIP was 0.456, whereas the posterior estimate of additive genetic variance ($\sigma_{g}^{2}$) on the observable scale was higher (0.844). The reason for lower variation at the liability scale is the logarithm transformation of the location parameters of both Poisson and negative Binomial distribution.

The mean posterior estimate of *h*^2^ was 0.320 with a posterior standard deviation of 0.162. For ticks in sheep there is, to date, only a published estimate of *h*^2^ for body louse (*Bovicola ovis*) in Romney sheep. The *h*^2^ for body louse score (log_e_[louse score +1]) varied from low to moderate, depending on the time of measurements, i.e., January 2002: *h*^2^ = 0.13, March 2002: *h*^2^ = 0.17 and November 2004: *h*^2^ = 0.37 [56]. The genetic parameters for tick count in sheep are still lacking in the literature. In cattle, the *h*^2^ of tick count has been estimated and range widely; from low estimates of *h*^2^ (0.11 to 0.13) for the cattle tick *Boophilus microplus* in crossbred cattle [57,58] to moderate and high estimates of *h*^2^ (0.17 to 0.42) [59–61]. Our estimate of *h*^2^ for tick count is in the range of the previous studies in sheep and cattle, indicating that accuracy in predicting genetic effects for tick count is relatively high and the tick load can be reduced through selective breeding.

The estimate of *h*^2^ for tick count in both sheep and cattle varied due to different statistical models and trait definitions. For the trait definition, we used the sum of tick counts from three positions, which are very likely to be in contact to ticks during grazing season. With this approach, it may not represent the total number of ticks on each lamb as applied in the other studies. Yet, variation of ticks in the hot spots *per se* should still reflect the variation in susceptibility or a general robustness to tick. Counting ticks of the whole body is more labour-intensive and time consuming than just counting at only three positions. Hence, a future study should quantify the genetic correlation between tick counts from these three positions and the whole body to verify if they can be considered the same trait for which it may be selected using a selection index.

In addition, the results proved that the maternal care may be a very important factor that determines the variation in tick count. The mean of maternal environmental variance over **McMC** replicates (Fig 2) was high ($\sigma_{m}^{2}$ = 0.183) and even higher than $\sigma_{sd}^{2}$. We did not quantify maternal genetic variance but in cattle, it has shown to be very low (0.004 or only 2% of phenotypic variance). The maternal environmental variance may also reflect that the ewes take their lambs to different parts of the pastures with different levels of tick infestation. Maternal care is a temporary environmental factor that can be improved and taken into account by farming management to reduce tick infection.

### Response to selection

**Fig 3:**
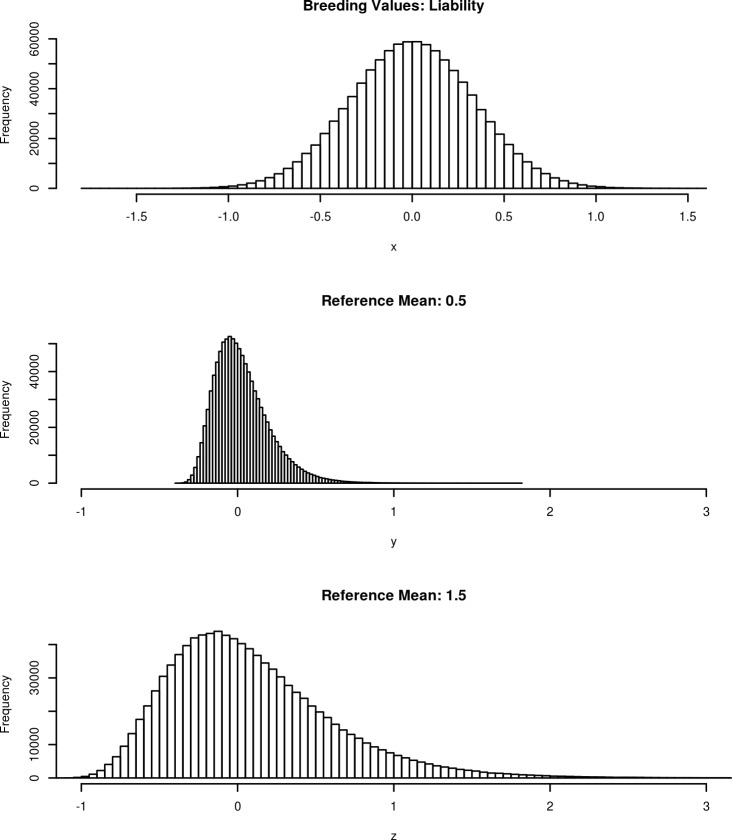
Distribution of simulated breeding values for tick counts on liability scale and on the observed scale at different reference means (0.50 and 1.50).

In order to quantify the relevance of the genetic variation in a practical breeding scheme, we calculated the expected number of tick count for the top and bottom individuals of the population. The results are presented in Table 3. Given the non-linear nature of the model, the expected selection response will depend on a reference point defined by the trait mean. Thus, a selection of 5% downward would imply a reduction of ticks from 0.064 to 0.383 depending on the reference point (0.5 to 3 ticks) and a selection of 5% upward will increase the number of ticks from 0.084 to 0.502. The asymmetry of selection response is also associated with the non-linear nature of the model, which is coherent with the theoretical predictions of Foulley [42]. Thus, even when the breeding values were symmetric in the log scale the response of the observed scale is asymmetric. To illustrate this fact, we present the distribution of the simulated breeding values given the posterior mean estimate of $\sigma_{sd}^{2}$ in the ZIP model on the observed scale with reference means of 0.5 and 1.5 (Fig 3).

**Table 3:** Expected response per generation (on logarithm and observes scales) to upward and downward selection on whole population for tick infestation.

Method	Selection rate	log-scale		Trait mean					
			0.5	1.0	1.35	1.5	2.0	2.5	3.0
Upward	5%	0.161	0.084	0.167	0.226	0.251	0.335	0.418	0.502
	10%	0.108	0.055	0.109	0.147	0.164	0.219	0.273	0.328
	20%	0.064	0.032	0.063	0.085	0.095	0.127	0.158	0.190
Downward	20%	-0.052	-0.024	-0.048	-0.065	-0.073	-0.097	-0.121	-0.146
	10%	-0.100	-0.045	-0.091	-0.123	-0.136	-0.182	-0.228	-0.273
	5%	-0.143	-0.064	-0.127	-0.172	-0.191	-0.255	-0.319	-0.383

Given the mean of tick count of this studied population of 1.350, the reduction of the breeding values on the liability in one sire-dam genetic standard deviation (*σ*_*sd*_ = 0.337), will imply a reduction of the number of ticks of approximately 0.37 leading to an average tick count lower than 1. Such expected genetic gain will considerably reduce the need for treatment with insecticide and thereby likely reduce the incidence of tick-borne infections.

To apply a selective breeding program, a trait needs to be routinely measured. This could imply a challenge as counting tick is labour intensive. Furthermore, to get accurate tick counts, sheep farmers cannot use acaricides. This is still not practically feasible at most commercial sheep farms with tick-infested pastures. In addition, the maternal environmental effects may be important as the ewes’ care of their lambs may be a crucial factor determining the number of ticks on their lambs. Future studies may also investigate maternal genetic effects on tick-count to be included in a selection index. However, a more efficient system to record the trait has to be developed to select accurately. The use of genomic selection may unravel the genetic potential of this trait and enables the ability to predict breeding values without the need to record phenotypes of selection candidates on a routine basis [62]. Thus, genomic selection may provide more accuracy to selection on maternal traits. However, knowledge about the genetic architecture of this trait is needed to design a breeding scheme using genomic selection against tick load in lambs.

## Conclusions

Zero-inflated Poisson model is the most parsimonious for fitting tick count data. Genetic variation and heritability estimates for number of ticks on Norwegian lambs suggest potential for improving tick resistance through selective breeding. A reduction of the breeding values by one sire-dam genetic standard deviation on the liability scale may reduce the number of tick counts below an average of 1. The maternal care may be an important environmental factor affecting the number of ticks on lambs, and should be accounted for. Further, knowledge on the correlation between tick infestation and risk of developing tick-borne disease needs to be established.

## Supporting information

S1 Table LogCPO and DIC from four different models. (PDF)
