# Supplementary material for: A comparison of nonlinear mixed models and response to selection of tick-infestation on lambs
Source: PLoS One. 2017 Mar 3;12(3):e0172711. doi: 10.1371/journal.pone.0172711 (PMC5336382; doi:10.1371/journal.pone.0172711)
Supplement: S1 Table — (PDF) [file pone.0172711.s001.pdf]

Supporting Information

S1 Table. LogCPO and DIC from four different models*.

Model	LogCPO	DIC	κ	η
ZINB	-1440.5	2815.0	10644.74 (2793.89)	0.042 (0.016)
ZIP	-1440.0	2812.4	n.a.	0.042 (0.016)
NB	-1448.5	2866.3	69.29 (295.56)	n.a.
P	-1464.9	2827.6	n.a.	n.a.

*sire-dam genetic variance was fixed to be zero. ZINP = zero-inflated negative binomial model, ZIP = zero-inflated Poisson model, NB = negative binomial model, P = Poisson model. LogCPO= logarithm of conditional predictive ordinate, DIC = deviance information criterion, κ = shape parameter of negative binomial distribution, η = probability of zero-inflated model. n.a. = not applicable.
